# Dopamine D3 Receptor Heteromerization: Implications for Neuroplasticity and Neuroprotection

**DOI:** 10.3390/biom10071016

**Published:** 2020-07-09

**Authors:** Federica Bono, Veronica Mutti, Chiara Fiorentini, Cristina Missale

**Affiliations:** 1 Division of Pharmacology, Department of Molecular and Translational Medicine, University of Brescia, 25123 Brescia, Italy; v.mutti002@unibs.it (V.M.); chiara.fiorentini@unibs.it (C.F.); mariacristina.missale@unibs.it (C.M.); 2 “C. Golgi” Women Health Center, University of Brescia, 25123 Brescia, Italy

**Keywords:** dopamine, dopamine D3 receptor, nicotinic acetylcholine receptor, heteromerization, neuroplasticity, neuroprotection, bivalent ligands

## Abstract

The dopamine (DA) D3 receptor (D3R) plays a pivotal role in the control of several functions, including motor activity, rewarding and motivating behavior and several aspects of cognitive functions. Recently, it has been reported that the D3R is also involved in the regulation of neuronal development, in promoting structural plasticity and in triggering key intracellular events with neuroprotective potential. A new role for D3R-dependent neurotransmission has thus been proposed both in preserving DA neuron homeostasis in physiological conditions and in preventing pathological alterations that may lead to neurodegeneration. Interestingly, there is evidence that nicotinic acetylcholine receptors (nAChR) located on DA neurons also provide neurotrophic support to DA neurons, an effect requiring functional D3R and suggesting the existence of a positive cross-talk between these receptor systems. Increasing evidence suggests that, as with the majority of G protein-coupled receptors (GPCR), the D3R directly interacts with other receptors to form new receptor heteromers with unique functional and pharmacological properties. Among them, we recently identified a receptor heteromer containing the nAChR and the D3R as the molecular effector of nicotine-mediated neurotrophic effects. This review summarizes the functional and pharmacological characteristics of D3R, including the capability to form active heteromers as pharmacological targets for specific neurodegenerative disorders. In particular, the molecular and functional features of the D3R-nAChR heteromer will be especially discussed since it may represent a possible key etiologic effector for DA-related pathologies, such as Parkinson’s disease (PD), and a target for drug design.

## 1. Introduction

Dopamine (DA), one of the main neurotransmitters in the central nervous system (CNS), controls several physiological functions related to locomotor activity, learning and memory, cognition, attention, affective behavior, motivation and reward and endocrine regulation. DA also modulates a variety of functions in the periphery, including catecholamine release, cardiovascular function, renal function, vascular tone, hormone secretion and gastrointestinal motility [1].

DA exerts its effects by binding to and activating specific G protein-coupled receptors (GPCR) that represent the largest superfamily of cell surface receptors targeted by different classes of drugs. In mammals, five subtypes of DA receptors have been identified, labeled D1 through D5. These receptors are classified into two families based on structural, pharmacological and signaling properties. The D1-like family consists of D1 and D5 receptor subtypes (D1R and D5R), while the D2-like family comprises the D2, D3 and D4 receptors (D2R, D3R and D4R). Each receptor displays unique properties, including affinity to DA, and shows a peculiar neuronal distribution [1]. Interestingly, increasing evidence suggests that DA receptors can diversify and amplify their repertoire of signaling by forming homo- and hetero-dimers, a property typically shared by the GPCR family [2] that greatly increases their heterogeneity.

The relevance of DA is such that dysfunctions of DA transmission and receptor signaling are implicated in many neuropsychiatric disorders, including attention deficit hyperactivity disorder (ADHD), schizophrenia, psychosis, Tourette Syndrome (TS) and depression, and in neurodegenerative diseases, including Parkinson’s disease (PD), Huntington disease (HD) and multiple sclerosis (MS) [3]. Moreover, aberrant DA transmission underlies drug addiction [4]. On this line, modulation of DA transmission can control the symptoms of many diseases and DA receptors are important targets for drug discovery.

The D3R is expressed on DA neurons, both at the somatodendritic level and at synaptic terminals, in the substantia nigra (SN) and ventral tegmental area (VTA), as well as in the ventral striatum [5,6,7]. D3R are also found in the islands of Calleja and cerebellum [6,7] and, at low density, in medium spiny neurons (MSN) of the ventral [8] and dorsal striatum [7,9,10,11]. Activation of D3R modulates a variety of functions, including rewarding and motivating behavior [12], some features of cognitive functions [13] and locomotor activity [14]. Pre-synaptic D3R have been classically considered autoreceptors inhibiting both DA neuron firing and DA release [9,15,16]. Interestingly, D3R is characterized by high affinity for DA (420-fold higher than that of D2R); moreover, unlike D2R, small changes in the number or function of D3R severely affect synaptic transmission, a characteristic suggesting that this receptor could play a relevant role as a modulator of physiological dopaminergic function [13]. Moreover, there is substantial evidence that the D3R exerts neurotrophic, neuroprotective and neurorestorative effects on DA neurons. On this basis, a new and essential role for D3R-mediated neurotransmission has been suggested, both in preserving DA neuron homeostasis in physiological conditions and in counteracting neuronal alterations prodromal to neurodegeneration [17,18,19,20,21,22,23].

Given the emerging role of D3R to the etiology and treatment of specific neurodegenerative and neuropsychiatric diseases, here, we discuss structural and functional aspects of the D3R including its interactions with other receptors, with a particular focus on the mechanisms involved in DA neuron protection.

## 2. Structure and Function of D3 Receptor

The D3R was first described and characterized in 1990. This receptor displays marked sequence homology and in vitro ligand-binding similarity with the D2R [1,24] and this characteristic made it hard to develop compounds specifically acting on D3R. This similarity also represented one of the major impediments to the characterization of D2R and D3R as well as to the elucidation of their role in the pathogenesis of DA-related disorders. Some years ago, however, the identification of the crystal structure of the D3R and the deduced homology model of the D2R structure highlighted differences that can be exploited for drug design [25]. Among them, an allosteric binding site, extending toward the extracellular loop 2 of the D3R, has been identified that may be crucial in determining ligand selectivity [25]. As a member of the D2-like receptor family, the DR3 is coupled to the Gαi/o protein, which, by inhibiting adenylyl cyclase (AC), blunts cAMP formation and protein kinase A (PKA) activation [1,26,27,28]. More recently, it has been demonstrated that the D3R also modulates other transductional pathways, such as the extracellular signal-regulated kinases 1/2 (Erk1/2) cascade, an effect that could be important for different D3R-mediated cellular events, such as morphological differentiation, synaptic transmission and neuronal plasticity [21,29]. Moreover, several data indicate that the D3R also promotes activation of the Akt pathway through the phosphoinositide 3-kinase (PI-3K) [30,31], leading to the activation of the mammalian target of rapamycin (mTOR), one of the main signaling pathways involved in neuron survival and structural plasticity [32,33,34]. In DA terminals, the D3R, as the D2R, regulates extracellular DA concentrations [15,35] by modulating DAT redistribution from intracellular compartments to the cell surface, an effect that requires both Erk1/2 and PI3K activation [30]. This variety of signaling transduction contributes to D3R functional heterogeneity since different pathways may be activated in specific brain regions and, more importantly, specific signaling mechanisms could be dysfunctional in pathological conditions.

Recently, a substantial progress in understanding the functional aspects of the D3R has been made by studying the neurodegenerative process. Increasing evidence indeed suggests a unique role of the D3R in neuroprotection. In particular, in experiments carried out in animal models, intraventricular infusion of (±)-7-hydroxy-N,N-di-n-propyl-2-aminotetralin (7-OH-DPAT), a D3R-preferring agonist, promotes neurogenesis in the rat SN [36]. Moreover, in a 6-OH-DA lesion-based rat model of PD, chronic administration of 7-OH-DPAT restores the dopaminergic nigrostriatal pathway [23], an event consistent with stimulation of neurite outgrowth and increased DA release. We have recently reported that the D2-like receptor agonists quinpirole and 7-OH-DPAT exert neurotrophic effects on DA neurons in primary mouse mesencephalic cultures, as shown by increased maximal dendrite length, number of primary dendrites and soma area [21]. These effects required the activation of the PI-3K-Erk1/2 and PI-3K-Akt-mTOR pathways [31,34]. These results suggest that the structural plasticity of DA neurons is substantially promoted by the D3R, a conclusion supported by the finding that the neurotrophic effects of D2-like agonists were abolished by the specific D3R antagonist SB2770121A and were vanished in DA neurons derived from D3R-KO mice [21,31]. By using human-induced pluripotent stem cells (hiPSCs)-derived neurons form healthy subjects, we recently provided a widespread characterization of D3R expression and function, throughout the differentiation process, from hiPSCs toward the acquisition of a phenotype characteristic of authentic DA neurons [18]. In particular, we showed that the mRNA encoding for the D3R was already detected in the hiPSC stage and that the D3R is involved in the amplification of the multipotent/neuronal progenitor cell population before the acquisition of a finally differentiated DA phenotype. These data are consistent with previously published data showing the prenatal expression of the D3R in rodents [37,38,39] and in human embryonic stem cells [40]. We have also shown that, in addition to its classical role as an autoreceptor [1,18], stimulation of D3R promotes neurotrophic effects [18,41] and plays a crucial role in triggering key intracellular events with neuroprotective potential [18,19].

Taken together, these data suggest that the D3R may be crucially involved in the control of DA neuron trophism during development [18,39,42], a property also shared with the D2R [43,44], and in counteracting early pathological events that may subsequently result in neurodegeneration [18,21,22,23].

## 3. D3 Receptor Heteromerization

As with the majority of GPCR, DA receptors were classically considered to operate as monomers that interact with G proteins to modulate specific effectors. However, in the last two decades, several GPCRs have been shown to directly interact with other receptors to form homodimers, heterodimers or high-order oligomers [45] and, among them, DA receptors appear to be highly promiscuous proteins able to form heterodimers. Dimerization can involve the extracellular loops, as in the case of the m3 muscarinic receptor dimers [46], the transmembrane helices, as was described for the β2-adrenergic receptor dimerization [47], and the intracellular loops, as in the case of the GABA_B_ receptor dimerization [48], and both covalent [49] and non-covalent bonds can structurally stabilize heterodimers. Although the physiological function of heterodimers is not completely defined, it is well known that receptor heterodimerization may modify the ligand binding profile, the signaling transduction and the cellular trafficking of interacting receptors. The formation of receptor heterodimers may give rise to novel receptors units with unique pharmacological, signaling and trafficking properties that are different from those of their monomeric counterparts [50,51]. Intriguingly, heterodimers may be involved in cellular processes underlying several human disorders, not only in the CNS, but also in peripheral areas (for a review, see References [52,53]). Therefore, targeting specific GPCR heterodimers may represent a promising alternative to conventional drug development approaches.

The D3R may form heterodimers with other DA receptor subtypes, such as the D1R and the D2R [14,50,54] (Table 1). In particular, heterodimerization of D1R and D3R has been demonstrated in the striatum and nucleus accumbens (NAc) [54]. From a functional point of view, D1R-D3R heterodimerization increases the affinity of DA for the D1R and the potency of DA in activating AC via the D1R and impairs agonist-induced D1R internalization [14,54], suggesting that, within the D1R-D3R heterodimer, D1R-mediated transmission is likely potentiated by the D3R. Moreover, a synergistic cross-talk between D1R and D3R agonists in activating Erk1/2 signaling has also been described [55]. More recently, it has been reported that the simultaneous activation of D1R and D3R within the D1R-D3R heteromer results in G protein-independent, ß-arrestin-dependent Erk1/2 and Akt activation both in the NAc and in transfected cells [56]. The characteristics and localization of the D1R-D3R heterodimer in the striatum suggest that this complex could be the functional unit mediating the development of levodopa (L-DOPA)-induced dyskinesia in PD models [52,57,58,59,60], thus providing a unifying mechanism for D1R- [52,61,62] and D3R-mediated alterations in the development of these side effects of L-DOPA therapy.

In the CNS, co-localization of D2R and D3R has been reported both in DA neuron synaptic terminals, and in post synaptic dopaminergic projections, mostly in the globus pallidus and NAc [6], and indication of physical interaction between these receptors has been provided [50,63]. Many drugs, including D2-like receptor agonists, show high potency and efficacy at the D2R-D3R heterodimer, suggesting that this receptor complex could potentially play a role in the pathophysiology and treatment of several brain diseases [63]. Beside its interaction with DA receptor subtypes, the D3R may also form complexes with other GPCRs (Table 1). Specifically, it has been reported that adenosine A2AR and D3R interact to form the A2AR-D3R heterodimer, in which the A2AR antagonistically modulates both the affinity and the signaling of the D3R [64]. Moreover, on the basis of pharmacological and functional studies, an interaction has been proposed for the neurotensin NTS2 receptor and D3R [65], and for the endothelin ETB receptor and D3R [66], even if the existence of these heterodimers has not been conclusively demonstrated. More recently, heterodimers containing the D3R and either the MT1 or MT2 melatonin receptors have been detected in both transfected cells and in human eye postmortem tissues, where they are thought to regulate intraocular pressure. This heterodimerization abolishes D3R-Gi coupling and signaling to the Erk1/2 pathway [67]. Beside GPCRs, other structurally and functionally different classes of receptors, as ion channel receptors, may interact with D3R [68] (Table 1). In particular, we recently reported a direct interaction of D3R with the nicotinic acetylcholine receptor (nAChR) [20].

Taken together, these findings indicate that the pharmacological and functional characteristics of the D3R may be specifically modulated in different brain areas or in pathological conditions by interactions with other membrane receptors.

## 4. Interaction of D3R and nAChR: Relevance for Neuroplasticity and Neuroprotection

The activity of DA neurons is regulated by an integrated interplay between different proteins and receptor systems. Among them, the DA transporter (DAT), that is responsible for DA reuptake, plays a central role in the regulation of spatial and temporal functions of DA [70,71], while D2R and D3R autoreceptors are crucial in inhibiting DA neuron firing and DA release [15,35]. Moreover, several heteroreceptors, including the nAChR, are involved in the control of DA transmission.

### 4.1. The Nicotinic Acetylcholine Receptor

nAChR, that are widely expressed in the nervous system, are composed by five subunits, symmetrically organized to form a central pore, permeable to Na^+^, K^+^, and, to varying degrees, to Ca^2+^. Up to now, nine alpha (α2–α10) and three beta (β2–β4) subunits, have been identified [72] and several studies showed that the association of different α and β subunits generates multiple receptor subtypes. In particular, the α7 subunit has a high propensity to form homomeric nAChRs [73], even if it can also interact with the b2 subunits to form heteropentamers [74,75,76]; by contrast, the α2–α6 subunits are incapable of forming homomeric receptors and require the β subunits for the formation of functional heteropentameric nAChRs [77,78]. The α4β2* (asterisk indicates possible presence of other subunits in the receptor) and α7 nAChR subtypes are the most expressed in the CNS [78,79]. Heterogeneity in subunit composition contributes to differences in nAChR functional properties, such as ion permeability and desensitization. In particular, homomeric α7 receptors are known to desensitize rapidly and to have a high Ca^2+^ to Na^+^ permeability ratio. As a result, activation of α7 nAChR channels can impact on several Ca^2+^-dependent mechanisms, including activation of second messenger pathways [77]. On the other hand, heteropentamers, like the α4β2 nAChR, are characterized by an especially high permeability to Na^+^ and K^+^, resulting in cell excitation. This excitation may activate voltage gated calcium channels allowing calcium influx into the cell. These nAChR subtypes are widely distributed in the brain and are especially localized both in synaptic terminals and in somatodendritic compartments [80]. One of the primary functions of nAChR is to modulate synaptic transmission and synaptic plasticity triggered by other neurotransmitters, resulting in alterations in affective behavior, attention and cognition [81]. Midbrain DA neurons express high levels of nAChR [82], especially those containing the α4 and/or α6 subunits and the β2 subunit. In particular, the α6β2 and α4β2 subtypes are mainly expressed in DA nerve terminals and the α4β2 is the prevalent subtype in somatodendritic compartments [73,83]. Moreover, recent evidence has undoubtedly identified the α6-containing nAChR as the key subtype mediating the effects of nicotine in the NAc; on the other hand, in the dorsal striatum, the predominant nAChR are those containing the α4β2 subunits [83]. As a consequence, targeting α6 or non-α6 nAChR could differentially modulate behavioral reinforcement or sensorimotor function.

### 4.2. D3–nAChR Interactions

The effects of nicotine on DA release have been extensively studied in the NAc, since α6β2 nAChR play a major role in the reinforcing effect of this compound [84]. On the other hand, an inverse correlation between cigarette smoking and PD development has been documented [85], an observation that may be related to nicotine-induced structural plasticity and protection on DA neurons [85,86]. These observations thus underlie the important role of the nicotinic system in the control of DA transmission in both physiological and pathological conditions. Since nAChR and both D2R and D3R are extensively co-localized in both DA neuron cell bodies and nerve terminals, the possibility that functional interactions may occur between DA receptors and nAChR has been investigated and clearly demonstrated [87]. A cross-talk between D2R and nAChR in DA terminals has been reported, with nAChR-mediated stimulation of DA release being inhibited by the D2R [18,88]. This antagonistic cross-talk could be easily explained by the formation of a nAChR–D2R complex, since a physical interaction between β2* nAChR and D2R has been suggested by co-immunoprecipitation experiments [88]. Since D2R, D3R and nAChR are co-localized in different DA neuron domains, it is likely that D2R, D3R and nAChR might be associated into different heteromeric complexes that may exert strong control over DA release and DA neuron viability. Nicotine, in fact, beside controlling DA release, activates neuroprotective signals in DA neurons, supports DA neuron regeneration and modulates the expression of different genes regulating neuronal morphogenesis [19,89,90].

**Figure 1:**
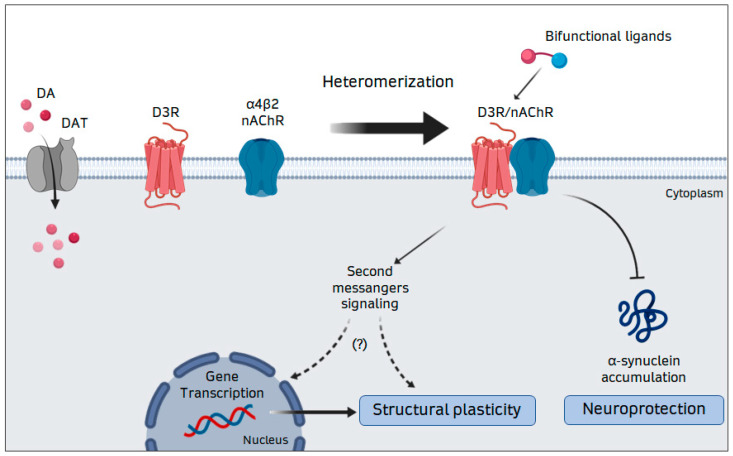
Representation of the heteromerization between the D3R and the α4β2 nAChR in DA neurons. Abbreviations: DA, dopamine; DAT, dopamine transporter; D3R, dopamine D3 receptor; nAChR, nicotinic acetylcholine receptor.

We have recently reported that nicotine provides neurotrophic support to DA neurons by increasing their dendritic arborization and soma size [18,34]. This effect is mediated by the α4β2 nAChR subtype and depends on functional D3R, since it was blocked by D3R-preferential antagonists and was absent in DA neurons from D3R-KO mice [34]. These observations suggest the existence of a functional cross-talk between D3R and nAChR in promoting DA neuron trophism. By using Bioluminescence Resonance Energy Transfer (BRET), we demonstrated that the D3R directly interacts with the β2 subunit of nAChR to form a heteromeric complex [20]. Moreover, by using the proximity ligation assay (PLA), we identified the D3R-nAChR heteromer in cultured DA neurons and mouse mesencephalic brain sections [20], as well as in hiPSCs-derived DA neurons [19]. Interestingly, disruption of D3R-β2 interaction by a cell-permeable interfering peptide abolished the effects of nicotine on DA neuron morphology [20], suggesting that the D3R-nAChR heteromer represents the molecular unit triggering nicotine-mediated neurotrophic effects (Figure 1). The D3R-nAChR heteromer is also involved in neuroprotection of DA neurons. By using glucose deprivation (GD)-induced neurotoxicity, we reported that both D3R agonists and nicotine modulate alpha-synuclein (alpha-syn) accumulation and protect DA neurons against neuronal injury [17,18]. More recently, we reported that nicotine inhibits alpha-syn accumulation in hiPSCs-derived DA neurons, an effect that was specifically blocked by D3R antagonists [18] and was lost in the presence of the specific interfering peptide [19], suggesting that, beside the induction of neurotrophic effects, the D3R-nAChR heteromer is the molecular unit involved in neuroprotection and inhibition of alpha-syn accumulation (Figure 1). Taken together, these data suggest that, by modulating neuronal functions such as excitability, synaptic plasticity and structural plasticity, the D3R-nAChR heteromer likely controls DA signaling in different brain areas, thus representing a possible key etiologic effector for DA-related pathologies, such as PD. More importantly, these observations suggest that alterations in the assembly and function of this receptor complex, by impairing the neurotrophic and neuroprotective support to DA neurons, may result in early dysfunctions contributing to the specific vulnerability of DA neurons [91].

## 5. Therapeutic Implications of D3R-nAChR Heteromerization

Development of compounds acting on DA receptors has been the main approach for the treatment of disorders of DA transmission, including PD and schizophrenia. However, these drugs are associated with side effects, making it crucial to identify new targets for the development of alternative therapeutic strategies. On this line, the concept of GPCR heteromerization, raising different combinatorial possibilities, is likely to have a profound impact on drug discovery. The use of a single compound able to simultaneously modulate multiple targets has been, in fact, proposed. Theoretically, such ligands would bind to the two components of the heterodimers, and thus incorporate two pharmacophores. These drugs, called bifunctional ligands, can be broadly defined, in fact, as a molecule that contains two discrete recognition units linked through a spacer [92]. In particular, bifunctional ligands that are selective for GPCR heterodimers may offer fundamental therapeutic advantages. The properties of a given GPCR may be different from cell to cell depending on the cellular environment and the relative expression of other receptors. Thus, molecules that interact and activate/inactivate specific heterodimers may be an attractive therapeutic tool. First, these ligands should target only tissues expressing both interacting receptor protomers, potentially reducing the incidence of side effects; second, bifunctional ligands offer the advantage to detect native heterodimers in vivo. Since several GPCR heterodimers have been identified in heterologous cell systems, the existence of these receptor complexes needs to be validated in a physiological context before becoming a real therapeutic target. Strategies to develop novel bifunctional ligands for receptor heterodimers have been reported [93,94]. On the other hand, it is possible that many known ligands may show either substantial heterodimer selectivity or may modulate heterodimer formation and function. For example, among the reported bifunctional compounds, BIM-23A387, acting on the heterodimer composed by the somatostatin subtype 2 receptor (sst2) and the D2R, inhibits GH release from pituitary tumors with higher potency than individual receptor agonists either given alone or in combination [95]. Moreover, the opioid agonist 6′-guanidinonaltrindole has the unique property of activating heterodimers containing delta opioid receptor (DOR) and kappa opioid receptor (KOR), but not the corresponding homodimers. Since spinal cord neurons co-express DOR and KOR, probably as heterodimers, 6′-guanidinonaltrindole may represent an analgesic drug selectively acting at the spinal cord level with reduced side effects [96,97].

Heteromers formed by D3R and nAChR might represent the target of bifunctional compounds that, by maximizing the neurotrophic effects of D3R and nAChR, may be potentially useful for treatment of PD. For instance, D3R agonists, such as pramipexole and ropinirole, have been widely used to treat motor deficits of PD [98,99]. However, to date, no intervention using DA agonists has shown efficacy or is designed as being useful for slowing PD progression. In particular, clinical studies using ropinirole [100] and pramipexole [101] failed to support the earlier suggestions of disease-modifying effects of these drugs [100,101,102]. On the other hand, clinical trials using nicotine, as a neuroprotective drug, are still ongoing [103]. Our data suggest that the D3R-nAChR complex could be a molecular target for drugs with disease-modifying activity in PD patients [19,104].

Compounds endowed with a dual agonist profile at the target receptor complex should, in fact, exert a synergistic neurotrophic effect, since the neurotrophic properties of nicotinic agonists [18,34,89] as well as of D3R agonists [18,21,41,105] have been documented. Molecular prototypes with this bivalent mechanism of action should be pivotal to validate the D3R-nAChR complex as an unprecedented therapeutic target for PD and could pave the way for developing original drug candidates in light of a novel pharmacological intervention on the disease. On this line, we have already synthesized a small set of ligands, which incorporate in the same structural skeleton pharmacophoric elements, enabling interaction with both nAChR and D3R units. These compounds belong to the family of bivalent ligands [106], which have gained interest as potentially innovative therapeutic agents [107,108]. These probes have been designed connecting, by means of a partially rigidified spacer, the compound A-84543, a selective α4β2 nAChR agonist, to ropinirole, a D3R preferential agonist. Although in each of these compounds, the spacer chain varies in terms of length, degree of rigidity and functionalization, all the compounds bind with high affinity to both β2-subunit-containing nAChR and D3R [104]. Interestingly, among all these bivalent ligands, the compound named HyNDA-1, characterized by the shortest linker, has been selected for its remarkable characteristics. We reported, in fact, that HyNDA-1 significantly promoted neurotrophic remodeling of both mouse and human DA neurons, an effect prevented by either nAChR or D3R antagonists [104]. Moreover, disrupting the D3R-nAChR heteromer by the specific interfering peptide [20] counteracted the neurotrophic effects of HyNDA-1 [104]. Interestingly, by using the BRET assay, we also found that HyNDA-1 increases the affinity of interaction between D3R and nAChR in the HEK-293 transfected cell system [104], reflecting the property of HyNDA-1 of bridging the two interacting receptors. Taken together, these results may suggest that this novel bivalent compound may represent the framework to develop new drug candidates, since the discovery of compounds with disease-modifying features, such as the capability to block neurodegeneration or to promote neuroregeneration/neurogenesis, is an urgent need for the cure of PD.

Targeting the D3R-nAChR heteromer could also be relevant for other DA-related disorders, in particular, nicotine addiction. Tobacco smoking is one of the main public health threats and represents a social and economic problem. The addictive properties of nicotine are mediated by its interaction with the nAChRs expressed in mesolimbic DA neurons. This neuronal population represents the main reinforcement circuit of the brain, where they stimulate the release of DA, that is also under the inhibitory control of D2R and D3R autoreceptors. Although nAChRs are the initial sites of nicotine in the brain, downstream events involving dopaminergic reward pathways may be critical in reinforcing smoking effects. In particular, a growing body of evidence suggests that the D3R is involved in the mechanisms of drug dependence and abuse, pointing to the potential use of selective D3R receptor antagonists for the therapeutic management of relapse to drug-seeking behaviors [109,110,111,112,113].

The D3R-nAChR heteromeric complex would be a potentially innovative target to be considered for smoking cessation. Available tobacco-use cessation drugs have targeted the α4β2 nAChR. For example, one of the first-line therapies for smoking interruption is varenicline, a partial agonist for α4β2 nAChR. Varenicline stimulates basal mesolimbic dopamine release to approximately 50% of the maximal effect of nicotine, inhibits nicotine-induced dopamine release and reduces nicotine self-administration [114,115,116]. Interestingly, being this compound targeted to the α4β2 nAChR, the possibility cannot be excluded that it could also interact with the D3R-nAChR heteromer. However, this possibility remains to be investigated.

A tentative synthesis of putative bifunctional derivatives designed by connecting a β2-containing nAChR antagonist with a D2R/D3R agonist moiety has recently been performed [117]. In this case, the structural feature of the selective β2-containing nAChR antagonist was those of N-n-alkyl nicotinium salts, a class of potent and competitive nicotinic antagonists. On the other hand, a group of 2-(alkilaminomethyl) chromanes has been chosen to form the moiety with a selective D2R/D3R agonist profile. The four synthetized compounds combined the pharmacological profile of their individual pharmacophores, in other words, as antagonists at nAChR and as an agonist at D2R/D3R. One of these compounds showed the most intriguing properties since it has high affinity at the β2-containing nAChR and low efficacy as a D2R/D3R agonist, thus representing one of the first attempts at the identification of selective bifunctional ligands that, acting through the D3R-nAChR heteromer, may induce a marked reduction of DA release [117].

All together, these data suggest that novel bifunctional compounds characterized by a dual agonistic profile, due to their neurotrophic properties, could find a potential application in the treatment of PD. On the other hand, bifunctional nicotinic antagonist-D2R/D3R agonist compounds should ideally inhibit DA release, a condition that could be exploited in therapeutic protocols for the control of nicotine addiction.

## 6. Conclusions and Future Directions

In conclusion, the data summarized here show that the D3R interacts with other GPCR as well as with structurally and functionally different families of receptors, to form heterodimers with peculiar pharmacological, signaling and trafficking properties that may be different from those of the individual interacting receptors. Identifying receptor heterodimers and decoding their characteristics is a critical step for understanding their contribution to the pathogenesis of psychiatric and neurological disorders. Moreover, heterodimers may represent the targets for drug discovery. Of particular interest is the heterodimer composed by the D3R and β2 subunit-containing nAChR, a new receptor entity playing a unique role in supporting DA neuron growth and survival. Dysfunctions of both D3R and nAChR have been reported to play a role in the development of several disorders, including PD and addiction, and the discovery of the D3R-nAChR heterodimer may open the way to the design and development of novel drugs capable to positively modulate this heterodimeric complex, to support DA neuron plasticity and survival and to protect DA neurons from toxic damage.

It should be noted, however, that many aspects of the D3R-nAChR complex remain to be deeply investigated. In particular, the influence of the D3R-nAChR complex in the establishment of complex behavioural phenotypes should be fully elucidated. Thus, detailed studies in animal models are necessary to define the D3R-nAChR heteromer brain distribution and function in vivo. In particular, by using interfering peptides that disrupt the D3R-nAChR heteromer formation [19,20], as well as genetically modified animals that lack one of the interacting receptors, it will be possible to define unique functional properties of the D3R-nAChR heteromer. In addition, it will be crucial to investigate the expression and function of the D3R-nAChR heteromer in animal models of specific diseases, including PD and addiction. Finally, the development of new bifunctional compounds specifically targeting the D3R-nAChR heteromer needs to be implemented.

## Figures and Tables

**Table 1:** Heteromeric complexes containing the D3R.

Receptor Complex	Detection Method	Suggested Relevance
DA receptors		
D1R–D3R	FRET and BRET in transfected cell lines [14,54]	Parkinson’s disease [14,54,62]
	CO-IP in transfected cell lines and rat striatum [54]	Addiction [56,62]
	PLA in rat and monkey striatum [69]	LID [54,56,62,69]
D2R–D3R	CO-IP in transfected cell lines [50]	Parkinson’s disease [51,63]
		Schizophrenia [51]
Other GPCRs		
D3R–A2AR	FRET in transfected cell lines [64]	Parkinson’s disease [64]
		Schizophrenia [64]
D3R–MT1R/MT2R	BRET in transfected cell lines [67]	Intraocular pressure [67]
	PLA in human non-pigmented ciliary body epithelial cells [67]	
Ion Channels		
D3R-nAChR	BRET in transfected cell lines [20]	Parkinson’s disease [19,20]
	PLA in mouse primary mesencephalic DA neurons and midbrain sections [20], and in hiPSC-derived DA neurons [19]	Addiction [20]

Abbreviations: A2AR, adenosine A2A receptor; BRET, bioluminescence resonance energy transfer; CO-IP, co-immunoprecipitation; DA, dopamine; D1R, dopamine D1 receptor; D2R, dopamine D2 receptor; D3R, dopamine D3 receptor; FRET, fluorescence resonance energy transfer; GPCRs, G-protein coupled receptors; LID, L-DOPA-induced dyskinesia; MT1R/MT2R, melatonin receptor 1 and 2; nAChR, nicotinic acetylcholine receptor; PLA, proximity ligation assay.
